# Development of InDels markers for the identification of cytoplasmic male sterility in *Sorghum* by complete chloroplast genome sequences analysis

**DOI:** 10.3389/fpls.2023.1188149

**Published:** 2023-07-17

**Authors:** Myeong-Eun Choe, Ji-Young Kim, Rizwana Begum Syed Nabi, Sang-Ik Han, Kwang-Soo Cho

**Affiliations:** Department of Southern Area Crop Science, National Institute of Crop Science, Rural Development Administration, Miryang, Republic of Korea

**Keywords:** *Sorghum bicolor*, chloroplast genome, CMS, phylogenetic tree, InDel

## Abstract

Cytoplasmic male sterility (CMS) is predominantly used for F1 hybrid breeding and seed production in *Sorghum*. DNA markers to distinguish between normal fertile (CMS-N) and sterile (CMS-S) male cytoplasm can facilitate F1 hybrid cultivar development in *Sorghum* breeding programs. In this study, the complete chloroplast (cp) genome sequences of CMS-S and Korean *Sorghum* cultivars were obtained using next-generation sequencing. The *de novo* assembled genome size of ATx623, the CMS-S line of the chloroplast, was 140,644bp. When compared to the CMS–S and CMS-N cp genomes, 19 single nucleotide polymorphisms (SNPs) and 142 insertions and deletions (InDels) were identified, which can be used for marker development for breeding, population genetics, and evolution studies. Two InDel markers with sizes greater than 20 bp were developed to distinguish cytotypes based on the copy number variation of lengths as 28 and 22 bp tandem repeats, respectively. Using the newly developed InDel markers with five pairs of CMS-S and their near isogenic maintainer line, we were able to easily identify their respective cytotypes. The InDel markers were further examined and applied to 1,104 plants from six Korean *Sorghum* cultivars to identify variant cytotypes. Additionally, the phylogenetic analysis of seven *Sorghum* species with complete cp genome sequences, including wild species, indicated that CMS-S and CMS-N contained *Milo* and *Kafir* cytotypes that might be hybridized from *S. propinquum* and *S. sudanese*, respectively. This study can facilitate F1 hybrid cultivar development by providing breeders with reliable tools for marker-assisted selection to breed desirable *Sorghum* varieties.

## 1 Introduction

*Sorghum* (*Sorghum bicolor* [L.] Moench) is the fifth most important major cereal cultivated worldwide and it is used not only for human nourishment, but also for animal fodder and feed, construction material, fencing, and brooms (Doggett, 1988; Mundia et al., 2019). *Sorghum* is a diploid C4 plant with outstanding tolerance to most types of abiotic stress (Tari et al., 2013). *Sorghum’s* genome is significantly smaller genome than maize (around 800 vs. 2,500 Mb), and it has recently undergone high-quality diploid genome sequencing, making it an emerging model for highly productive C4 crops (Paterson et al., 2009; Mccormick et al., 2018). Heterosis, or hybrid vigor, is the ability of hybrids to outperform elite inbred line parents and is probably the most important strategy to increase grain yield in various crops, including *Sorghum* (Kim and Zhang, 2018). In several vegetable and cereal crops, commercial seed production is based on F1 hybrids produced using the cytoplasmic genetic male sterility (CMS) system. The CMS system relies on a set of male sterility-inducing cytoplasm that are complemented by alleles at genetic loci in the nuclear genome that either restore fertility or maintain sterility. In 1952, a milo CMS cytoplasm was identified in the offspring of a hybrid between two cultivars, *milo* and *kafir*, wherein *milo* as the female and *kafir* as the male. The A1 CMS line sourced from *milo* is the predominant CMS line used to produce hybrids in *Sorghum*. The presence of restorer genes enabling the production of fertile F1 hybrids using the CMS approach is essential for the cost-effective production of hybrid *Sorghum* seeds. To date, a total of nine distinct resources of CMS, namely, A1 (*milo*), A2, A3, A4, Indian A4 (A4M, A4VZM, and A4G), A5, A6, 9E, and KS cytoplasm, have been identified in *Sorghum* (Schertz, 1977; Li and Li, 1998). Although most grain *Sorghum* types currently utilized in agricultural production are hybrids in the world, hybrid cultivars are still not widely used in Korea, which explains the low average yields.

The chloroplast is the primary site of photosynthesis and carbon fixation in plants, is an essential organelle for land plants (Daniell et al., 2016), and is inherited maternally (Asaf et al., 2017). Some cp genome sequences have been used to distinguish between species and conduct phylogenetic studies, as the chloroplast (cp) genome is more conserved and shorter in length than the nuclear and mitochondrial (mt) genomes. The cp and mt genomes are often used to study plant evolution (Cho et al., 2015; Lu et al., 2016; Hong et al., 2017; Hong et al., 2019). Although the cp genome’s specific structure and sequences remain conserved, the larger mt genome (200−2400 kb) has a significantly different structure and various isoforms, even within a single plant cell (Sugiyama et al., 2005; Cho et al., 2022). Therefore, polymorphic DNA originating from the cp genome is favorable for developing CMS markers, even though several CMS genes are mitochondrial as they are maternally inherited (Cho et al., 2006).

The mt and cp genomes of the normal fertile (CMS-N) of *S. bicolor*, BTx623, have previously been sequenced and are under the GenBank accession numbers NC_008360 and NC_008602, respectively. Recently, ergonomically important genes, such as those of the flowering time (Casto et al., 2019), dwarf (Hilley et al., 2017) and brown midrib (Bout and Vermerris, 2003) have been isolated using genetic mapping and comparative genomic studies between *Sorghum* and other crops. The mt genome rearrangement between CMS-S and CMS-N in *Sorghum* was analyzed, and it was discovered that the coding region of the *coxI* gene in CMS9E was found to be extended at the 3’- end by 303 nucleotides, resulting in an extension of 101 amino acids at the C-terminal of the protein. A novel chloroplast DNA deletion has been reported in most CMS lines of *Sorghum* and this deletion occurred in the middle of the gene *rpoC2*, coding for the *ß”*-subunit of RNA polymerase (Bailey-Serres et al., 1986; Chen et al., 1993). Chen et al. (1995) also reported that *rpoB*, *rbcL*, and *rpoC2* transcripts are low in inflorescence tissues and pollen of CMS. Molecular characterization of the cytoplasm using mitochondrial DNA probes revealed sufficient diversity to broaden the cytoplasmic base of *Sorghum* hybrids (Xu et al., 1995; Sivaramakrishnan et al., 1997). A previous study demonstrated the strict maternal inheritance of mt and cp DNA in the *Sorghum* cytoplasm (Pring et al., 1982). All genes encode proteins with a mitochondrial transit peptide and numerous penta-tatricopeptide repeats (Kante et al., 2018; Praveen et al., 2018). The cytoplasmic male sterile line (S *rfrf*) and its near-isogenic maintainer line (S or N *RfRf*) are essential for breeding F1 hybrids using CMS systems. The test cross is the most popular traditional method to identify the cytoplasmic type in *Sorghum*. DNA markers have been used for the indirect selection of major cultivation traits that distinguish the fertile and sterile individuals in several crops, such as onion, maize, wheat, cotton, and others (Bosacchi et al., 2015; Melonek et al., 2021).

This study aimed to: (i) Obtain complete chloroplast (cp) genome sequences of CMS-S and Korean *Sorghum* cultivars using next-generation sequencing, (ii) Identify single nucleotide polymorphisms (SNPs) and insertions and deletions (InDels) in the cp genomes that can serve as DNA markers for breeding, and phylogenetic studies, (iii) Develop InDel markers, including tandem repeats, to accurately distinguish between cytotypes (CMS-S and CMS-N) based on copy number variation, and validate their effectiveness in identifying cytotypes in Korean *Sorghum* cultivars.

## 2 Materials and methods

### 2.1 Plant materials and genome information

One male sterile line (ATx623) and four Korean cultivars of *S. bicolor* were used for the complete cp genome sequencing (**Table 1**). Five pairs of *S. bicolor* near-isogenic lines (male sterile and maintainer lines) and 1,104 individual plants from six Korean cultivars were used to identify cytoplasmic types with insertion and deletion (InDel) markers (**Tables 2**, **3**). To conduct comparative genome analysis, the cp genome sequence information in *Sorghum* species was retrieved from the National Center for Biotechnology Information (**Table 1**). All plants were grown at the Department of Southern Area Crop Institute in Miryang, Korea.

**Table 1:** List of *Sorghum* species and GenBank accession numbers of complete chloroplast genome sequences.

Species	Cultivars	GenBank Accessions	Genome size (bp)	Remark
***Sorghum bicolor***	BTx623	EF115542	140,754	Saski et al. (2007),
	ATx623	MT459453	140,644	Complete chloroplast genome sequence was *de novo* assembled in this study
	Nampoongchal^z^	MT333847	140,753	
	Donganme^z^	MT333845	140,644	
	Sodamchal^z^	MT333848	140,644	
	Hwanggeumchal^z^	MT333846	140,753	
***S. sudanense***		MH926028	140,755	Song et al. (2019)
***S. propinquum***		MH926027	140,642	Song et al. (2019)
***S. timorense***		KF998272	140,629	Song et al. (2019)
***S. halepense***		LS398105	140,810	GenBank(NCBI)
***S. arundinaceum***		LS398103	140,821	GenBank(NCBI)
***Hemisorghum mekongense***		KY596136	140,765	Arthan et al. (2017)

^z^Korean Sorghum cultivars developed by line selection.

**Table 2:** Identification of cytoplasmic male sterile factors in *Sorghum bicolor* using chloroplast specific insertion and deletion (InDel) markers.

No.	Lines	Genotypes	Cytoplasmic male sterile factor	Amplicon sizes of InDel markers (bp)	
				cp_01	cp_02
**1**	ATx630	Male sterile line (S*rfrf*)	S	270	265
**2**	BTx630	Maintainer line(N*rfrf*)	N	242	243
**3**	ATx631	Male sterile line (S*rfrf*)	S	270	265
**4**	BTx631	Maintainer line(N*rfrf*)	N	242	243
**5**	ATx2928	Male sterile line (S*rfrf*)	S	270	265
**6**	BTx2928	Maintainer line(N*rfrf*)	N	242	243
**7**	A03017	Male sterile line (S*rfrf*)	S	270	265
**8**	B03017	Maintainer line(N*rfrf*)	N	242	243
**9**	A.arg-1	Male sterile line (S*rfrf*)	S	270	265
**10**	B.arg-1	Maintainer line(N*rfrf*)	N	242	243

**Table 3:** Application of cytoplasmic male sterile factors identification using the chloroplast genome specific markers (InDel cp_01) in the Korean *Sorghum bicolor* cultivars.

Cultivars	No. of individual plants			Remark		
	Total	S cytoplasm	N cytoplasm	Released Year	Bred by	Breeding methods
Nampoongchal	195	0	195	2015	RDA^z^	Pure line selection
Donganme	170	170	0	2015	RDA	Pure line selection
Sodamchal	236	236	0	2016	RDA	Pedigree
Hwanggeumchal	313	0	313	–	GARES^y^	Landraces
Bareme	95	95	0	2022	RDA	Pedigree
Noeulchal	95	0	95	2022	RDA	Pedigree
Total	1,104	501	603			

^z^Rural Development Administration.

^y^Gangwon-do Agricultural Research and Extension Services.

### 2.2 Extraction of DNA, sequencing and chloroplast genome assembly

DNA was extracted from approximately 100 mg of fresh leaf samples using the NucleoSpin Plant II Mini Kit (Macherey-Nagel, Germany), following the manufacturer’s instructions. The quality and quantity of the genomic DNAs were examined using agarose gel electrophoresis and a NanoDrop 8000 spectrophotometer (Thermo Fisher, USA). Total DNA was sequenced using an Illumina HiSeq 2000 (Illumina, San Diego, USA), and raw reads ranged from 1.9 to 2.7 Gb (**Supplementary Table 1**). The cp genome sequences were determined from the *de novo* assembly of low-coverage whole-genome sequences according to previous reports (Hong et al., 2017; Hong et al., 2019). In particular, trimmed paired-end reads (Phred score > 20) were assembled using CLC Assembly Cell Packages (ver. 4.2.1, https://www.qiagenbioinformatics.com/products/clc-assembly-cell/) using default parameters. The cp genome sequence contigs were selected from the initial assembly through the Basic Local Alignment Search Tool using the *S. bicolor* cp genome sequence as a reference (GenBank accession number: EF115542). Gaps and ambiguous sequences were manually adjusted using Sanger sequencing. PCR amplification and Sanger sequencing were performed to verify the four junction regions between the inverted repeats (IRs) and large single copy (LSC)/small single copy (SSC). The cp genome annotation was conducted using GeSeq (Tillich et al., 2017) with the reference sequences of *S. bicolor* from GenBank. The cp genome map was illustrated using the OGDraw software (Lohse et al., 2007).

### 2.3 Development of CMS specific markers and PCR amplification

Single nucleotide polymorphisms (SNPs) and InDels between the male sterile and maintainer lines were precisely identified using a variant calling process through MAFFT (Katoh and Standley, 2013). To amplify the InDel regions, 20 ng of genomic DNA was used in 20 µL PCR mixture comprising 2× TOP simple preMix-nTaq master mix (Enzynomics, Seoul, Korea) consisting of 0.2 U/µL of n-taq DNA polymerase, 3 mM of Mg^2+^, and 0.4 mM of each deoxynucleotide triphosphate mixture with 10 pmol of each primer. The primer sequences used are listed in **Table 4**. PCR was conducted in a thermocycler (Veriti, Applied Biosystems, CA, USA) using the following cycling parameters: 95°C (5 min); 35 cycles at 95°C (20 s), 55°C (20 s), and 72°C (1 min); and the final extension was conducted at 72°C (5 min). The PCR products were analyzed by capillary electrophoresis (QIAxcel Advanced System, Qiagen, Germany) following the manufacturer’s protocol. PCR products were purified with the Wizard SV Gel and PCR Clean-Up System (Promega, Madison, USA) and sequenced by direct sequencing in Bioneer Co. (Bioneer, Daejeon, South Korea). Sequences were aligned using ClustalW in MEGA 11.

**Table 4:** The information of primers used in this study for the identification of cytoplasmic male sterile factors in *Sorghum bicolor* using the chloroplast genome sequences between the male sterile (ATx623) and maintainer (BTx623) lines.

Primers	Marker type	Sequence (5’-3’)	Length (bp)	Tm	Amplicon size (bp)		Location
					ATx623	BTx623	
cp_01	InDel	AGAGACCCCGTTTACCCCTA	20	59.8	270	242	*rpoC2 – rps2*
		TTGTTCCGATGGAACCTTCT	20	59.5			
cp_02	InDel	CGTGTTTGAAATTTTGGGTCT	21	58.9	265	243	*cemA - petA*
		CGAGTCTGTTGTCATTCTACTGC	23	59.1			

### 2.4 Genetic distance and phylogenetic analyses

To investigate the phylogenetic position of *Sorghum* depending on the cytotype, we used eight complete cp genome sequences. Six complete cp genome sequences of the *Sorghum* species were retrieved from GenBank (**Table 1**). Phylogenetic analysis was conducted using the maximum composite likelihood model with 1,000 bootstrap replicates in MEGA 11 (Tamura et al., 2021). A phylogenetic tree was constructed using the neighbor-joining method (Tamura et al., 2004) with MEGA 11.

## 3 Results

### 3.1 Chloroplast genome assembly and characterization

**Figure 1:**
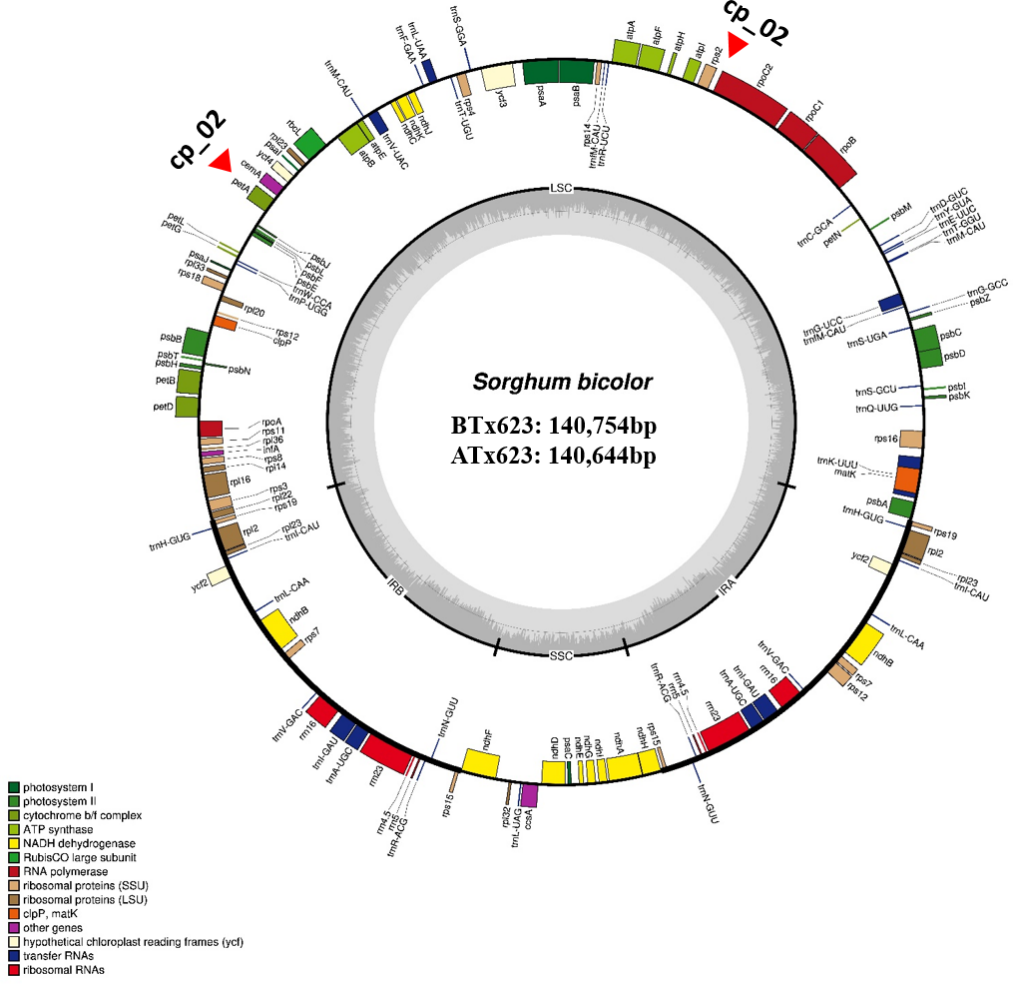
The complete chloroplast genome map of *Sorghum bicolor* in the male sterile (ATx623) and maintainer line (BTx623). The genes inside and outside the circle are transcribed in the clockwise and counterclockwise directions, respectively. Genes belonging to different functional groups are represented using different colors. Thick lines indicate the extent of the inverted repeats (IRa and IRb) that separate the genomes into small single-copy (SSC) and large single-copy (LSC) regions. The red-filled red triangles indicate the region of insertion and deletion (InDel) markers.

We sequenced and assembled the complete cp genomes of one isogenic line (CMS-S, ATx623) and four Korean *Sorghum* varieties using the Illumina HiSeq 2000 system. The complete sequences of the five cp genomes were generated using *de novo* and reference-based assemblies. Sequencing with approximately 1,657–20,373X coverage generated 130.28 Gbp of paired-end reads (**Supplemental Table 1**). The complete size of CMS-N is 140,754 bp, as reported by Saski (Saski et al., 2007). We found that the complete cp genome sizes of *S. bicolor* ATx23 and BTx623 were 140,644 and 140,754 bp, respectively and included a pair of IRs of 22,259 bp (CMS-N) and 22,782 bp (CMS-S) separated by SSC regions of 12,503 bp (CMS-N) and 12,506 bp (CMS-S) and LSC regions of 82,685 bp (CMS-N) and 82,574 bp (CMS-S) (**Figure 1**), respectively. The comparison of cp genomes of two inbred lines (ATx623 and BTx623) and four South Korean *Sorghum* cultivars (Nampoongchal, Donganme, Sodamchal, and Hwanggeumchal) showed no significant differences in their gene and gene order (**Figure 1** and **Supplementary Table 2**). All six cp genome structures had a typical quadratic structure.

A total of 103 genes were identified in the *Sorghum* cp genome, including 40 photosynthesis-related genes, 29 transfer RNA (tRNA) genes, and 4 ribosomal RNA (rRNA) genes. Sixteen genes contained one, two, or three introns, and six of these were tRNAs (**Supplemental Table 2**). Notably, six protein-coding genes (*rps12, rps15, rps19, rps7, rpl2*, and *rpl23*), eight tRNA genes (*trnA-UGC, trnH-GUG, trnI-CAU, trnI-GAU, trnL-CAA, trnN-GUU, trnR-ACG*, and *trnV-GAC*), and all rRNA genes were duplicated in the IR regions, which is common in most *Poaceae* genomes. The *Sorghum* cp genome contained 16 intron-containing genes. Among them, ten protein-coding genes (*petB*, *petD*, *atpF*, *ndhB*, *ndhA*, *rpoC1*, *rps12*, *rps16*, *rpl16*, and *rpl2*) and six tRNA genes (*trnA-UGC, trnG-UCC, trnI-GAU, trnK-UUU, trnL-UAA*, and *trnV-UAC*) had a single intron, and two genes (*rps12*, and *ycf3*) contained two introns (**Supplemental Table 2**).

Comparing the cp genome sequences of four *Sorghum* cultivars revealed that Nampungchal and Hwanggeumchal were near, whereas Donganme and Sodamchal were identical. This might be attributed to cultivar development from the same cytoplasmic background genetic resources. Phenotypically, these cultivars showed diverse traits, such as seed or grain color, lodging tolerance, culm length, plant height, and waxy endosperm. Thus, these cultivars could be important materials for further genetic research and new cultivar development.

### 3.2 Development and validation of CMS specific markers

**Figure 2:**
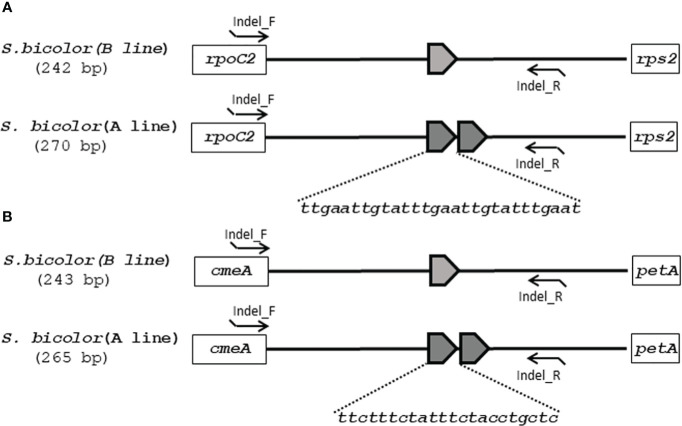
Schematic diagrams of **(A)** InDel cp_01 and **(B)** InDel cp_02 with cytoplasmic male sterile and maintainer lines of *Sorghum bicolor* in chloroplast genomes. **(A)** 28 and **(B)** 22 base pairs of tandem repeats (TRs) are represented as pentagons. The InDel cp_01 and InDel cp_02 marker primers are indicated as arrowed lines.

**Figure 3:**
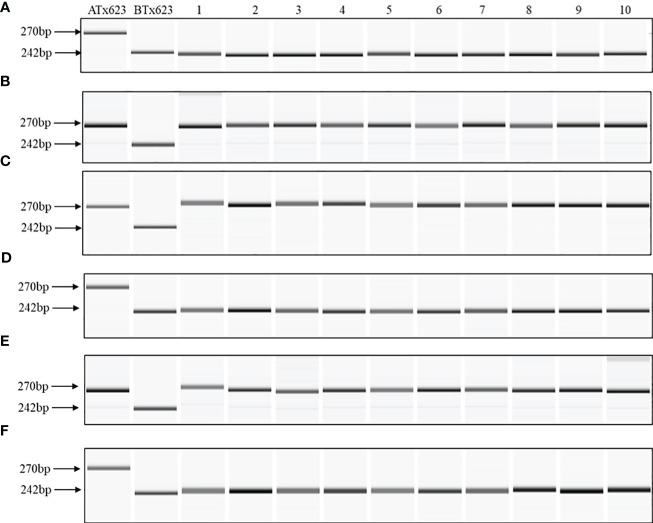
Capillary gel electrophoresis of Korean *Sorghum bicolor* cultivars with cytoplasmic male sterile factor-specific InDel cp_01 markers. **(A)** Nampoongchal, **(B)** Donganme, **(C)** Sodamchal, **(D)** Hwanggeumchal, **(E)** Bareme, and **(F)** Noeulchal. Ten individual plants (1 to 10) of each cultivar and cytoplasmic male sterile (ATx623) and maintainer (BTx623) lines are depicted.

Although the content and genes order in the cp genomes of the two *Sorghum* inbred lines and four South Korean *Sorghum* cultivars were very similar, numerous polymorphic sites were found among them. In the cytoplasm of cp genomes, 19 single nucleotide polymorphisms (SNPs) and 142 InDels in the genic region that can be used for marker development for breeding, population genetics, and evolution studies (**Supplementary Table 3**). When complete cp genomes were aligned to identify polymorphisms that may distinguish between S- and N-cytoplasms, wherein the difference is more than 20bp, we found that differences of 28 and 22 bp in the intergenic regions of *rpoC2-rps2* and *cemA-petA*, respectively, were due to those in copy number variation with two major tandem repeats (**Figure 2**). The alignment showed that InDels with lengths of 28 and 22 bp were present at the same position in the CMS-S cytotype (**Figure 2**). Thus, two pairs of primers were designed based on the InDels with length of 28 and 22 bp to amplify the regions (**Table 4**). To evaluate the accuracy of this marker, we tested five pairs of near isogenic lines (NILs) of *Sorghum* and compared the results with those obtained with the CMS specific InDel markers. PCR amplification showed that *Sorghum* cytotypes could be clearly distinguished using gel electrophoresis (**Figure 3**). All male sterile lines known to contain S-cytoplasm had upper bands of 270 and 265 bp with the cp_01 and cp_02 InDel markers, respectively. In contrast, all maintainer lines with N-cytoplasm had lower bands of 242 and 243 bp (**Table 2**). We also identified the cytoplasmic male sterile factors, namely, S or N, by InDel markers with 1,104 plants from six South Korean *Sorghum* cultivars (**Figure 3** and **Table 3**). The results of marker analysis revealed that individuals of each cultivar, Donganme, Sodamchalm, and Baremae, had 100% S-cytoplasm. In contrast, Hwanggeumchal, Nampungchal, and Noeulchal only contained N-cytoplasm types (**Table 3**). All cultivars containing S-cytoplasm were found to have a 28 bp insertion.

As *Sorghum* has a wide range of mating rates of more than 7-30% (Djè et al., 2000; Barnaud et al., 2008), we expected genetic variation in the cytoplasmic genome, but they were all identical. In *Sorghum* breeding, paper bags are used for repeated self-pollination and generation advancement to avoid outcrossing. Consequently, the developed varieties appear to have the same cytoplasmic type, implying that the breeding cultivars are genetically fixed, and the pure line is well maintained. Hwannggeumchal and Nampungchal contain the S-cytoplasm type, whereas Donganme and Sodmchal contain the N-cytoplasm type (**Table 1**). In *Sorghum*, the first report described a 165 bp deletion in the middle of *rpoC2* in CMS lines containing A1, A2, A5, and A6 cytoplasm (Chen et al., 1995). Consistent with previous results, all male sterile lines used in this study were included in the A1 cytoplasm.

In Korea, *Sorghum* is usually bred by landrace selection and utilization of landraces in a breeding program. The cytoplasm types in Korean *Sorghum* varieties have not yet been identified. Through cp genome sequencing, the cytotypes of Korean varieties, Hwanggeumchal and Nampungchal contained 140,753 bp, whereas those of Donganme and Sodamchal cytotypes contained 140,644 bp (**Table 1**).

### 3.3 Comparative chloroplast genome analysis with congeneric species of *Sorghum*

**Figure 4:**
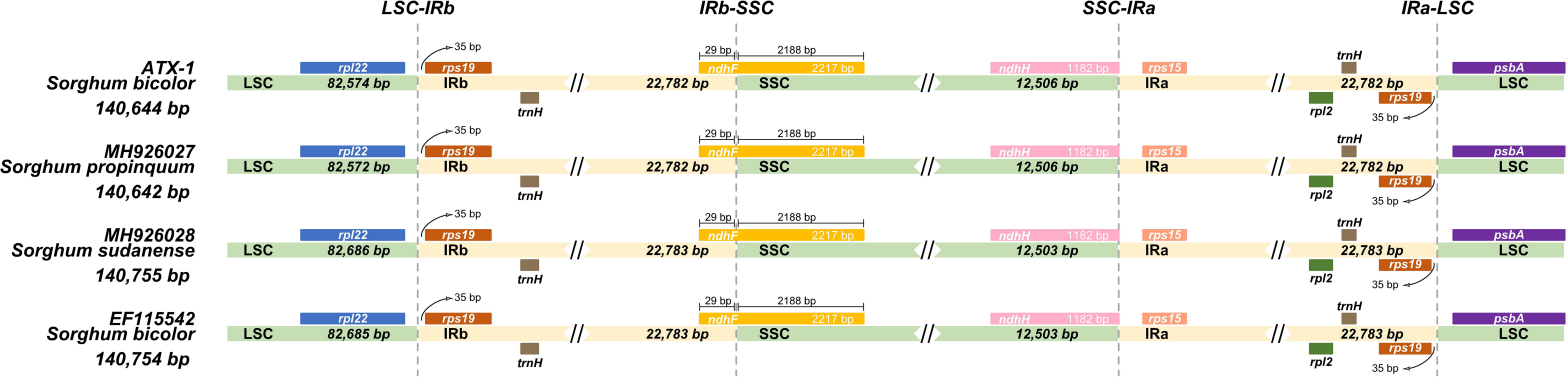
Comparison of the border position and size of LSC, SSC, and IR regions in the chloroplast genome of four Sorghum species. Gene names of each border are designated in boxes. LSC, Large Singe Copy; SSC, Small Single Copy; IRs, Inverted Repeats.

Typically, IR regions have identical lengths; however, they can extend or contract inside the chloroplast. Therefore, we compared the cp genomes of LSC, SSC, and IR (IRa and IRb) among ATx623*, S. sudanense, S. propinquum*, and BTx623 (**Table 5** and **Figure 4**). The total lengths of the cp genome of ATx623 and *S.propinquum* were nearly identical (140,644 and 140,642 bp, respectively), whereas those of *S.sudanens* and BTx623 were also identical (140,755 and 140,754 bp, respectively) (**Table 5**). However, the total cp genome lengths of *S. sudanense* and BTx623 were slightly longer (111-112 bp) than those of ATx623 and *S. propinquum* respectively. The guanine-cytosine content in the cp genome of *S. bicolor* (ATx623 and BTx623) and congeneric species (*S. sudanense* and *S. propinquum*) was 38.5%, and all the species contained 114 unique genes (**Table 5**). Additionally, we discovered that the size of intergenic spacers (IGSs) between the *rpl22-rps19* genes of *ATx623* in the junction between the LSC and IRb regions (LSC-IRb) were similar to those of *S. sudanense*, *S.propinquum* and BTx623 (**Figure 4**). Similarly, the IGSs between the *rps19- psbA* genes, located in the IRa-LSC junction, of ATx623*, S sudanense, S propinquum* and BTx623 were similar (**Figure 4**). The boundaries between the IRa regions were similar in size (1,182 bp) in all the compared species (**Figure 4**). Similarly, the *ndhH* gene spanned the IRb-SSC region, and the fragment located in the IRb region was equal in size (2,188 bp) among the compared species.

**Table 5:** Summary of chloroplast genome characteristics for four *Sorghum* genera containing the male sterile (ATx623) and maintainer line (BTx623) of *Sorghum bicolor*.

Category	*Sorghum bicolor* (ATx623)	*Sorghum propinquum*	*Sorghum sudanense*	*Sorghum bicolor* (BTx623)
GenBank accession No.	MT459453	MH926027	MH926028	EF115542
Total length (bp)	140,644	140,642	140,755	140,754
LSC length (bp)	82,574	82,572	82,686	82,685
SSC length (bp)	12,506	12,506	12,503	12,503
IRa length (bp)	22,782	22,782	22,783	22,783
IRb length (bp)	22,782	22,782	22,783	22,783
Total GC content (%)	38.48	38.48	38.49	38.49
Total number of genes	114	114	114	114

LSC, Large Single Copy; SSC; IR, Inverted Repeat.

### 3.4 Phylogenetic analysis

**Figure 5:**
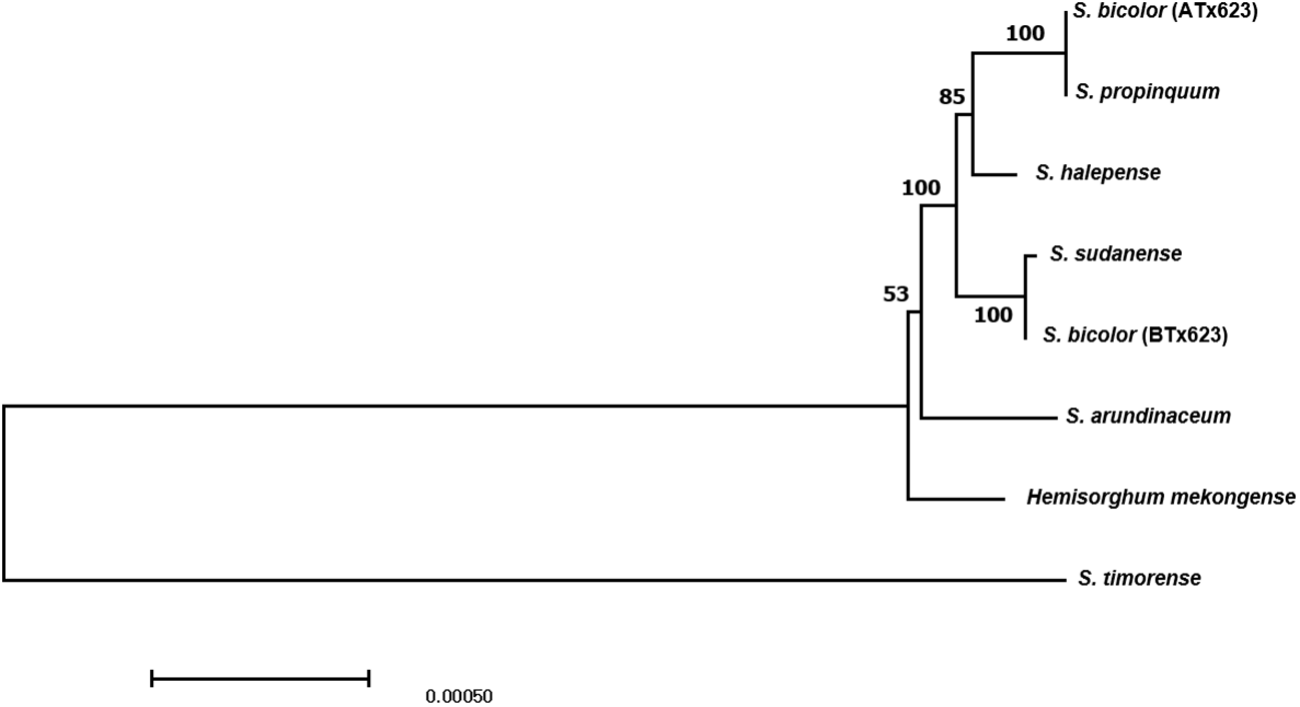
The phylogenic tree of six congeneric *Sorghum* species, including ATx623 (male sterile line) and BTx623 (maintainer line) of *Sorghum bicolor*, constructed using the maximum composite likelihood model. Bootstrap values are shown below each clade.

Molecular phylogenetic analysis offers new perspectives on the evolutionary linkages between species. Thus, phylogenetic analysis was conducted using the complete cp genome sequences of the eight *Sorghum* species. The results of the maximum composite likelihood analysis are shown in the phylogenetic tree (**Figure 5**). The phylogenetic tree was monophyletic and formed two clades within these eight *Sorghum* species, wherein *S. timorense* was the outgroup. A strong bootstrap value (100%) was observed for three of the five nodes. Eusorghum species, such as *S. bicolor* (ATx623), *S. propinquum, S. halepense, S. sudanense, S. bicolor* (BTx623), and *S. arundinaceum* were grouped into one clade, whereas *hemisorghum mekongense* formed another group clade. In the Eusorghum species clade, *S. biclor* (ATx623) was the sister to *S.propinquum* in the same branch, whereas *S. sudanense* was the sister to *S. bicolor* (BTx623), with a short branch length, indicating a dispersed evolutionary history or that they are a closer ancestor. Genetic distance analysis revealed the considerably less genetic distance between the analyzed *Sorghum* species. The lowest genetic distance value of 0.0000 was observed between *S. propinquum* and *S. color* (ATx 623) followed by the second lowest value of 0.00002 between *S. bicolor* (BTx623) and *S. sudanese* (**Supplementary Table 4**).

## 4 Discussion

The cp genome is a useful tool for analyzing the evolutionary relationships among species. This is due to the fact that photosynthesis-related organelles such as chloroplasts, contain a circular genome that is comparatively stable and is passed along from the mother to offspring. Moreover, recent research has focused on the cp genome, as it provides essential genetic information to investigate the evolutionary links between related species.

The overall structural organization and introns, genes, and gene order of the analyzed cp genome of a CMS-S line and South Korean cultivars of *Sorghum* were conserved and showed no significant difference in the cp genome size. Similarly, the SSC, LSC, IR regions, and GC content of cp genomes (38.5%) (**Table 5**) were also found to be similar among the Eusorghum species. These results are consistent with previous studies on the cp genomes of *Sorghum* and other species from the *Poaceae* family (Lu et al., 2016; Song et al., 2017; Song et al., 2019). Recently, numerous taxonomists have focused on the cp genome to investigate the phylogenetic relationships of related species. For example, cp genomes can provide sufficient genetic information for species identification. In this study, we developed InDel markers based on sequence variation in the cp genome for the accurate cytoplasm identification of species and developed InDel markers for further cytoplasm evaluation of species.

In the chloroplasts, the *ndhD* gene is a component of the NADH dehydrogenase complexes. The specific mutations in the *ndhD* gene hinder the NADH dehydrogenase complex’s ability to operate normally, which reduces the anther’s ability to produce energy. In wheat, a mutation in the *ndhD* gene causes male sterility in wheat. The mutation is a single-nucleotide substitution that changes a cytosine to a thymine results in a frameshift that leads to the production of a truncated *ndhD* protein (Han et al., 2022). *PsaA* and *psaB* gene are the subunit of photosystem I (PSI) and PSI complex of proteins that uses light energy to drive the transfer of electrons from water to NADPH. These complex has been shown to play a role in the regulation of gene expression in plants (Azarin et al., 2020). Therefore, we analyzed the *ndhD, psaA, psaB* gene sequences (nucleotide and amino acids) with Clutal W and we found that there is no genetic variation such as SNP or InDel between male sterile line and maintainer line (data not shown).

Recently, cp genome sequence analysis has been successfully used to reconstruct phylogenetic relationships among plant lineages. Previous phylogenetic studies based on entire cp genomes have been used to resolve the difficult phylogenetic relationships among closely related species. In this study, the whole cp genomes of a *Sorghum* CMS-S line and South Korean cultivars were sequenced and assembled using next-generation sequencing. In a previous study, four *Sorghum* species were grouped into two groups. *S. sudanense, S. bicolor*, and *S. propinquum* formed groups. *S. sudanense, S. bicolor*, and *S. propinquum* belong to the subgenus *Sorghum* which contains 10 species (Song et al., 2019). Phylogenetic analysis using the complete cp genome of seven *Sorghum* species, including wild species, revealed that CMS-S and CMS-N of the *S. bicolor* cytoplasm were highly similar to *S. propinquum* and *S. sudanense*, respectively. These results were consistent with those of a previous study (Song et al., 2019; Ananda et al., 2021). *S. propinquum* is a wild perennial diploid rhizomatous species distributed across Southeast Asia and the Indian subcontinent. Various traits are potentially useful for the introgression of *S. propinquum* into *S. bicolor*. The primary gene pool of modern *Sorghum* cultivars contains the wild species, *S. propinquum* (De Wet and Harlan, 1971; De Wet, 1978). Previous results found that S. *propinquum* showed increased height, early maturity, and high yield (Ananda et al., 2020). This might explain the close relationship between *S. bicolor* (ATx623) and *S*. *propinquum*.

In this study, the S cytoplasm of *S. bicolor Milo* was found to have genetic exchange with *S. propinquum*. In contrast, *S. sudanense* is believed to be segregated from a natural hybrid of *S. bicolor* and *S.arundinaceum*. Our findings revealed that *S. sudanense* is closely related to *S. bicolor*, which represents CMS-N, including the maintenance and restoration lines; hence, these results are consistent with those of previous studies. In *S. bicolor*, the *milo* cytoplasm (A1) has been widely used in hybrid production, and *kafir* has been used as a maintainer line as it produces fully fertile hybrids when crossed with the milo parent. These results phylogenetically support the fact that the *milo* and *kafir* cytotypes originated from *S. propinquum* and *S. Sudanese*, respectively. Bayesian inference analysis indicated that the *Sorghum* genus diverged from Miscanthus about 19.5 million years ago (mya). Smaller spikelets are a distinctive feature of *S. propinquum*. This is consistent with the morphology of the small anther, and pollen depleted exone caused by a 165 bp deletion of the *rpoC2* region, such as in the A1, A2, A5, and A6 cytoplasms. Doggett (1988) proposed that durra (*milo*) originated in Ethiopian because it contains the entire set of wild-type bicolor-durra crosses.

In the CMS system, the breeding programs were divided into two groups. One group was devoted to the development of the female inbred line (A/B-line) and the second was devoted to the development of the male inbred line (R-line). Prior to the hybrid development program, testcrossing, or sterilization, a new line should be identified as maintainers or restorers by a testcross. If the lines with the S cytoplasm have a dominant allele present in the nuclear genome, the plant will be an R-line to restore male fertility. Unless the line lacks the dominant allele for fertility restoration, the plant will be male-sterile (Senthil et al., 1994). Maintainer lines have the N cytoplasm and lack a dominant *Rf* allele. It is easy to discover a new B-line or predict the male infertility gene type by simply identifying the cytoplasmic type using a marker prior to crossbreeding. When developing B-lines, resources with the N-cytoplasm are first selected as markers, and new lines can then be cultivated through crossbreeding between B-lines. N (*Rfrf*) can also be used if the combinatorial ability test is conducted on a lineage with the N-cytoplasm. In conclusion, the newly developed InDel markers based on the cp genome variation can facilitate a new F1 hybrid breeding system in Korea.

## Data availability statement

The datasets presented in this study can be found in online repositories. The names of the repository/repositories and accession number(s) can be found in the article/**Supplementary Material**.

## Author contributions

M-EC conceived the design of the study, analyzed the data, and drafted the manuscript. J-YK and S-IH collected and grew *Sorghum* cultivars and lines in Miryang, Korea. RS conducted the bioinformatics work and was engaged in drafting the manuscript. K-SC was responsible for data analysis and writing of the manuscript. All authors read and approved the final manuscript. All authors contributed to the article and approved the submitted version.

## Supplementary material

Supplementary Figure 1 The comparison of the chloroplast genome structure of *Sorghum* genus using the mVISTA program.

Supplementary Figure 2 Multiple sequence alignment of InDel region in the chloroplast genome for the identification of cytoplasmic male sterile factors in *Sorghum bicolor*. (A) InDel cp_01 and (B) InDel cp_02. Each tandem repeat is shown as a black dotted line.
