# Supplementary figures and images for: Development of InDels markers for the identification of cytoplasmic male sterility in Sorghum by complete chloroplast genome sequences analysis
Source: Front Plant Sci. 2023 Jul 17;14:1188149. doi: 10.3389/fpls.2023.1188149 (PMC10388542; doi:10.3389/fpls.2023.1188149)

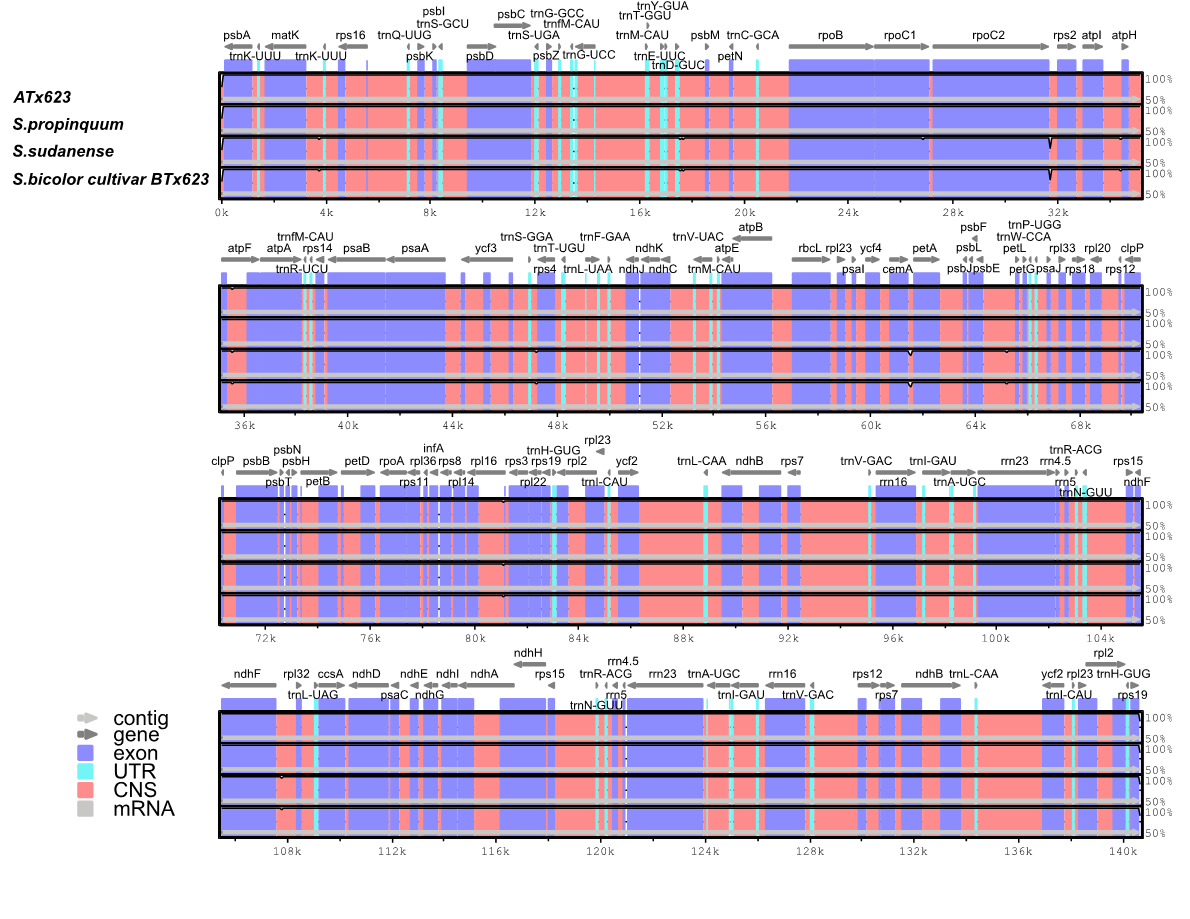

Supplement: Supplementary Figure 1 — The comparison of the chloroplast genome structure of Sorghum genus using the mVISTA program. [file Image_1.tif]

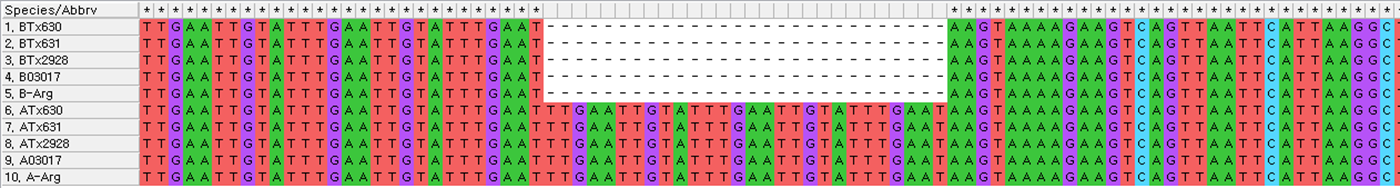

Supplement: Supplementary Figure 2 — Multiple sequence alignment of InDel region in the chloroplast genome for the identification of cytoplasmic male sterile factors in Sorghum bicolor. (A) InDel cp_01 and (B) InDel cp_02. Each tandem repeat is shown as a black dotted line. [file Image_2.tif]

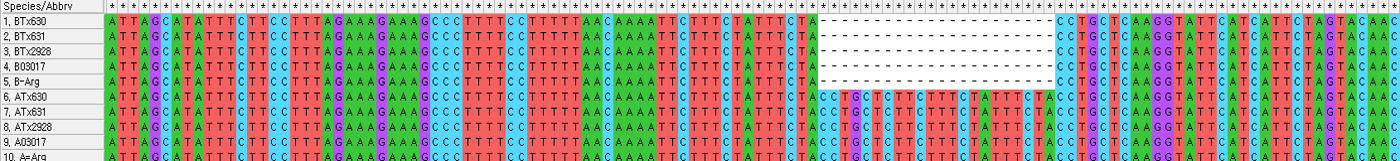

Supplement: Supplementary file 3 [file Image_3.tif]
